# Structural and Functional Differences between Homologous Bacterial Ribonucleases

**DOI:** 10.3390/ijms23031867

**Published:** 2022-02-07

**Authors:** Vera Ulyanova, Alsu Nadyrova, Elena Dudkina, Aleksandra Kuznetsova, Albina Ahmetgalieva, Dzhigangir Faizullin, Yulia Surchenko, Darya Novopashina, Yuriy Zuev, Nikita Kuznetsov, Olga Ilinskaya

**Affiliations:** 1 Department of Microbiology, Institute of Fundamental Medicine and Biology, Kazan (Volga Region) Federal University, 420008 Kazan, Russia; AlsINadyrova@kpfu.ru (A.N.); lenatimonina@rambler.ru (E.D.); akhmetgalievaa@bk.ru (A.A.); sokurenko.yulia@gmail.com (Y.S.); ilinskaya_kfu@mail.ru (O.I.); 2 Institute of Chemical Biology and Fundamental Medicine, Siberian Branch of the Russian Academy of Sciences, 630090 Novosibirsk, Russia; sandra-k@niboch.nsc.ru (A.K.); danov@niboch.nsc.ru (D.N.); nikita.kuznetsov@niboch.nsc.ru (N.K.); 3 Kazan Institute of Biochemistry and Biophysics, FRC Kazan Scientific Center of RAS, 420111 Kazan, Russia; dfaizullin@mail.ru (D.F.); yufzuev@mail.ru (Y.Z.)

**Keywords:** ribonuclease, binase, balnase, barnase, balifase, ribonuclease inhibitor, barstar, catalytic activity, structural organization

## Abstract

Small cationic guanyl-preferring ribonucleases (RNases) produced by the *Bacillus* species share a similar protein tertiary structure with a high degree of amino acid sequence conservation. However, they form dimers that differ in conformation and stability. Here, we have addressed the issues (1) whether the homologous RNases also have distinctions in catalytic activity towards different RNA substrates and interactions with the inhibitor protein barstar, and (2) whether these differences correlate with structural features of the proteins. Circular dichroism and dynamic light scattering assays revealed distinctions in the structures of homologous RNases. The activity levels of the RNases towards natural RNA substrates, as measured spectrometrically by acid-soluble hydrolysis products, were similar and decreased in the row high-polymeric RNA >>> transport RNA > double-stranded RNA. However, stopped flow kinetic studies on model RNA substrates containing the guanosine residue in a hairpin stem or a loop showed that the cleavage rates of these enzymes were different. Moreover, homologous RNases were inhibited by the barstar with diverse efficiency. Therefore, minor changes in structure elements of homologous proteins have a potential to significantly effect molecule stability and functional activities, such as catalysis or ligand binding.

## 1. Introduction

RNA molecules represent promising therapeutic targets for a multitude of important human diseases including infections and cancer. In recent years, a few RNA-targeted drugs that can modulate RNA functions have been identified [1,2]. Ribonucleases (RNases) play an important role in cell physiology by the regulation of RNA metabolism [3]. Catalyzing the degradation of RNA polymers, RNases indirectly participate in many cellular processes such as gene expression, cell growth and differentiation, and immune response [4,5,6]. The ability of RNases to inhibit the growth of tumor cells and the reproduction of viruses in infected cells was also shown [7,8]. The application of RNases as antitumor and antiviral agents implies their penetration into mammalian cells which contain a cytosolic RNase inhibitor (RI). RI regulates the activity of mammalian RNases and protects cellular RNA from their cytotoxic action [9]. Microbial RNases are insensitive to blocking action of RI [10]; thereby they are considered as perspective therapeutics.

One of the widely known bacterial RNases is binase from *Bacillus pumilus*, a member of the N1/T1/U2 family (IPR000026) [11]. It was shown that binase selectively kills different types of tumor cells and does not act on normal cells [12,13,14,15,16]. The protein has very low immunogenicity and does not induce a polyclonal T-cell response [12]. The study of the molecular mechanism of binase antitumor action has shown that the crucial role in the cytotoxicity of the enzyme belongs to its structural organization, catalytic activity and the participation in cell signaling by direct interaction with cell components [11,17].

RNases homologous to binase are produced by some other *Bacillus* species, such as *B. amyloliquefaciens* (barnase) [18], *B. altitudinis* (balnase) [19,20] and *B. licheniformis* (balifase) [21]. The bacillar RNases are small (~12 kDa) cationic proteins that share more than 73% identity of amino acid sequences, matching tertiary structures and close physicochemical properties. The active center of the RNases is formed by conserved residues and has a similar organization [22]. RNA hydrolysis is performed by these enzymes in two steps: the transesterification of the 5′-phosphodiester bond leads to the formation of nucleoside-2′3′-cyclophosphates as intermediate hydrolysis products, which are then cleaved to 3′-phosphates [23]. The RNases demonstrate preference to guanine when cleaving RNA [24]. The level of catalytic activity of the purified RNases is similar [19,21,25]. In *B. amyloliquefaciens* cells, the hydrolytic activity of barnase is controlled by the inhibitor protein barstar which is absent in *B. pumilus, B. altitudinis* and *B. licheniformis*; nevertheless, barstar can inhibit binase in vitro, though with a lower affinity than barnase [26,27].

Recent studies have shown that the quaternary structure of RNases plays a key role in the functional activity of the enzymes, particularly, catalytic properties, protein–protein interaction and subsequent biological effects [11,28]. The RNases of *Bacillus* form dimers in vivo [29] and higher order oligomers in vitro [30]. Despite the high degree of similarity between them, the mode of dimerization and stability of RNase dimers differ greatly, which can lead to functional differences between the homologous enzymes.

Here, we aimed to correlate the structural features of homologous RNases from *Bacillus* with their functional activities, such as the ability to interact with different RNA substrates and the inhibitor protein barstar. For the first time, the secondary structures of ribonucleases, their ability to form oligomers and hydrolyze different RNA substrates as well as to interact with barstar were characterized comparably.

## 2. Results and Discussion

### 2.1. Structural Characterization of Homologous RNases

Being close homologs, the guanyl-preferring ribonucleases from *Bacillus* have quite similar three-dimensional structures [11]. Despite this, only the amino acids of the active center of the RNases are identical [22]. Binase and balnase differ by a single amino acid substitution: threonine at position 106 in the binase is replaced by alanine in balnase, while binase and balifase has 73% identity of primary structures [21]. It is considered that structural divergence happens less rapidly than sequence divergence, therefore, structure-based alignments are supposed to be less informative for closely related proteins [31]. Conformational differences between homologous proteins occur often in the regions comprising turns and loops [32], which can lead to variance in their stability and interaction with other proteins [33].

**Figure 1:**
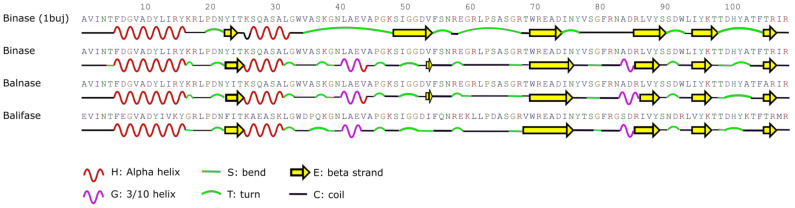
Distribution of structured regions along the RNase chains. Secondary structures of binase, balifase and balnase were predicted based on their amino acid sequences by DeepCNF. The elements of secondary structure of binase in solution (1buj) were extracted from PDB data.

To gain the precise details on local conformations in the RNase protein structures, we have predicted and compared secondary structure profiles of binase, balnase and balifase (Figure 1). It should be noted that out of these RNases, only binase has experimentally resolved the three-dimensional structure (PDB 1buj) which we have used as a reference. The DeepCNF algorithm has predicted 69% of residues as unordered and 1% as disordered conformation, in comparison to 15% α-helix and 14% β-strand in monomeric binase and balnase, whereas the monomer of balifase was described to have less coil and more β-strand positions (67% coils, 15% α-helix, 16% β-strand, 1% disordered) (Figure 1). As predicted, the balifase monomer has not only more β-strands, but also less exposed solvent accessible residues (35% exposed (E), 41% medium (M), 22% buried (B)) as compared with binase (42% E, 35% M, 22% B) and balnase (41% E, 36% M, 22% B).

The different secondary structures correspond to different solvent accessibilities of residues, with β-sheets being the most inaccessible structures, the helical conformation having an intermediate value, and random coils and turns being the most accessible folds [34,35]. The β-sheets are the most appropriate structures to shield the hydrophobicity of residues. The solvent accessibility of residues determines protein folding, stability and functioning [35]. The exposed residues are usually located on the surface of proteins and account for interactions with other proteins or ligands. The buried residues often form hydrophobic cores to maintain the conformation and structural integrity of proteins. Therefore, homologous RNases were predicted to have some differences in their secondary structures that can affect protein folding and oligomerization.

In all folds, there is a noticeable difference between the hydrophobic and hydrophilic residues. To represent the hydrophobicity value of a polypeptide, a grand average of hydropathicity index (GRAVY) is used. Its values vary between −2 to +2 for most proteins, with the positively rated proteins being more hydrophobic. We have calculated that the GRAVY index is more negative for balifase (−0.643) than for binase (−0.416) and balnase (−0.393), suggesting a higher level of its hydrophilicity. This is due to the increased number of lysine and glutamic acid residues in balifase (8 lysines and 6 glutamic acids) as compared to binase and balifase (5 lysines and 3 glutamic acids) which are the most accessible, irrespective of secondary structures. So, it seems that homologous RNases have some distinctions in the size of solvent accessible regions. It is proposed that such differences may affect protein–protein interactions and/or protein activity [36,37].

**Figure 2:**
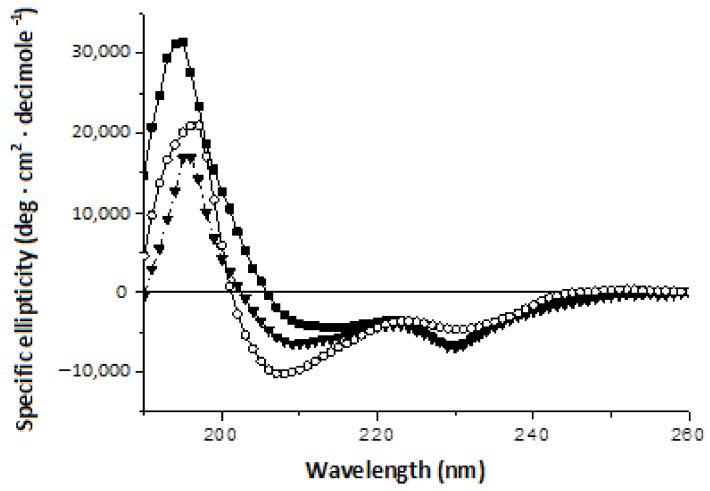
Circular dichroism (CD) spectral scans of binase (■), balnase (▼) and balifase (○).

To validate the predicted secondary structures of RNases, we have measured their circular dichroism (CD) spectra (Figure 2). The CD spectra of all RNases displayed an intense negative peak around 230 nm which is unusual for the absorption of purely peptide structures. Such a distortion of CD spectra is often observed in proteins with a high proportion of aromatic His, Phe, Tyr, and Trp residues, whose absorption extends to the far-UV range of spectrum [37]. As was previously shown, a minimum at 230 nm in barnase is associated with Trp35, Trp71 and Trp94 [38]. For such an effect to be realized, the certain spatial proximity and fixation of the aromatic ring in a specific position relative to peptide groups is necessary [38,39]. In other proteins the minimum at 230 nm was observed upon significant secondary structure rearrangement during protein aggregation [40]. Due to the mentioned distortion, the calculation of secondary structures from CD spectra by their approximating with canonical secondary structure spectra gives ambiguous results. Therefore, it is possible to estimate the differences in secondary structures of these RNases only approximately, comparing the shape and intensity of the undistorted part of the peptide spectra.

The obtained CD spectra have revealed that the short-wavelength minimum of ellipticity is quite wide in binase and falls at 216 nm, indicating an approximately equal contribution of β-structures and α-helices (Figure 2). In balnase, the visible minimum shifts to 209 nm, closer to the extreme of canonical helical structure of 208 nm, which suggests a larger proportion of helicity in this protein compared to binase. In balifase, if the depth of this minimum increases, its position shifts further from the short wavelengths, indicating an increase in the fraction of disordered structures. Thus, the performed analysis shows significant differences in the secondary structure of these three proteins, contrary to prediction.

The differences in secondary structures may account for differences in the folding and quaternary structure of homologous proteins. The differences in the CD spectra of homologous RNases are probably associated with distinctions in their quaternary structure. Different mechanisms of monomers binding are possible during the dimerization of RNases, resulting in various types of dimers [11]. At high protein concentrations, the association of different monomers results in domain swapping and the formation of stable oligomers. The domain-swapped form of barnase is derived from the same folding intermediate as its monomer [41]. Previously it was shown that the CD spectra of binase and its covalently cross-linked dimer differ in the content of secondary structure elements [42]. The content of β-structures decreases in dimer, due to the partial unfolding of the protein globule, while the number of α-helices remains unchanged [42].

**Figure 3:**
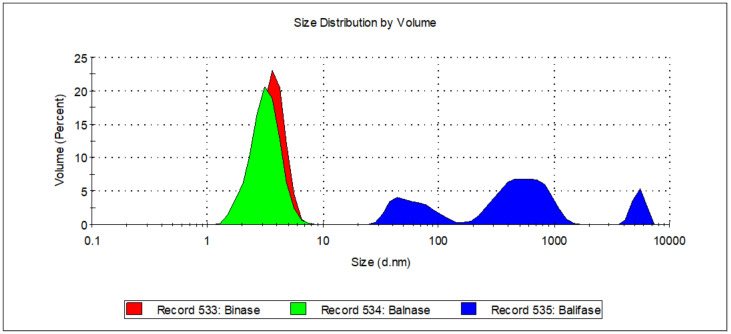
The size of RNase molecules in solution.

Guanyl-preferring RNases can form not only dimers of different conformations [11], but even trimers [41] and oligomers [30]. A mixture of multimers was earlier detected in barnase solution [43]. To assess the diversity of RNase forms we have performed dynamic light scattering experiments (Figure 3). This method is helpful to detect aggregates in macromolecular solutions, to determine the size of proteins and to monitor the binding of ligands. The hydrodynamic diameter of binase and balifase was found to be 3.67 nm and 3.25 nm, respectively (Figure 3), while the calculated size of their monomers is 1.6 nm [44]. Therefore, these RNases are represented in solution mainly by dimeric forms. On the contrary, balifase is represented by large particles (more than 62.9 nm) suggesting its oligomeric state.

We have also measured the zeta potential of RNases. It has shown that all of them have negative protein surface charges. Zeta potential values of binase, balnase and balifase were measured as −5.44 mV, −16.3 mV and −24.6 mV, respectively.

Thus, substitutions in the primary structures of homologous RNases mediate the differences in their secondary structure which may influence protein stability, folding and aggregation as was shown for some proteins [45,46,47].

### 2.2. Comparison of Catalytic Activities of RNases

Various types of RNA serve as a substrate for RNases and represent their main molecular target in the cell. It was shown that various dimeric forms of RNase A have differences in catalysis of RNA substrates [28]. Since bacterial RNases differ in the mode of their dimerization, their affinity to RNA substrates can be different. We have assessed catalytic activity of homologous RNases on natural RNA substrates: transfer RNA (tRNA), double-stranded RNA (dsRNA) and high-polymeric RNA (hpRNA). We have found that the RNases are active on all these substrates with efficiency decreasing in the row hpRNA >>> tRNA > dsRNA (Table 1). The level of specific activity of homologous RNases was approximately the same.

**Figure 4:**
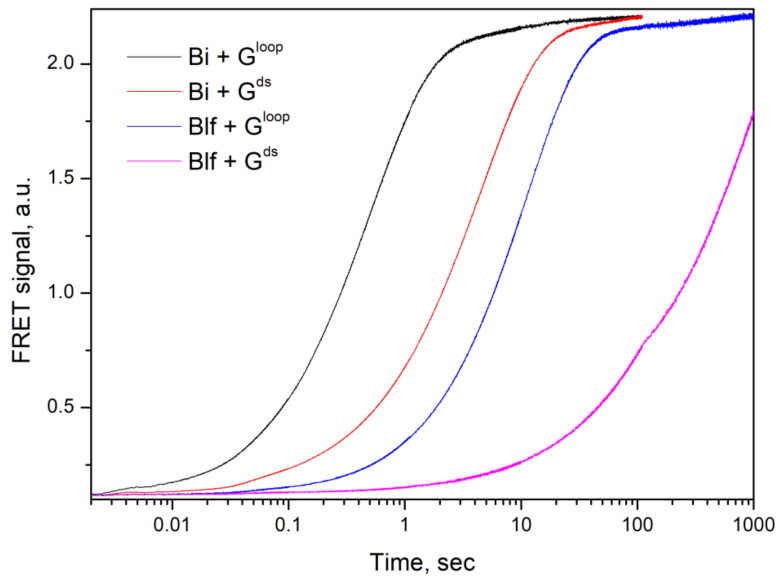
The change in the FAM fluorescence intensity during the interaction of binase (Bi) and balifase (Blf) with the G^loop^ and G^ds^ substrates. The enzyme and substrate concentrations were 0.5 µM.

We have compared kinetics of RNA cleavage by RNases on model substrates using the stopped flow technique. Considering the high degree of similarity between binase and balnase, which differ by only one amino acid at position 106, we have included only binase and balifase into the analysis. The model substrates were represented by short hairpins with a 6 bp stem and a 5 bp loop. Since the studied RNases are guanyl-specific on short-length substrates, the guanosine residue was placed at the loop (G^loop^-substrate) or the stem (G^ds^-substrate) (Table 2). Oligoribonucleotides contained the FRET-pair consisting of the fluorescein residue (FAM) and quencher (BHQ1). The interactions of binase and balifase with the substrates lead to an increase in FAM fluorescence intensity (Figure 4). The cleavage rate of the G^loop^ substrate was significantly higher than that of G^ds^ substrate for the both enzymes.

The initial phase of increase of FAM fluorescence intensity on the kinetic curves was used for the approximation of an exponential in Equation (1), which allowed calculating the values of the observed rate constants (Table 3). The rate constant value for the interaction of binase with a G^loop^ substrate was 9-fold higher than that of balifase. At the same time, the rate constant for the formation of a catalytic complex with the G^ds^ substrate was 30-fold higher for binase than for balifase. It should be noted that balifase was more sensitive to the location of the guanosine residue at the hairpin loop or stem. The rate constant value of the interaction of balifase with the G^loop^ substrate was 11-fold higher as compared to the G^ds^ substrate. As for binase, this ratio was about 3 (Table 3).

Thus, the homologous guanyl-preferring RNases are capable of cleaving diverse RNA substrates but with different rates and efficiencies. The ability to hydrolyze different types of RNA was shown for many RNases. RNase from frog *Rana pipiens* (onconase) degrades tRNA and dsRNA, α-sarcin (*Aspergillus* RNase) cleaves 28S rRNA and colicin E3 (*Escherichia coli* RNase) hydrolyzes 16S rRNA [13]. dsRNA is found among nuclear RNAs and some viral genomes. The ability to cleave dsRNA can endow RNases with the power of gene expression regulation and defense against viral infections. Transfer RNA accounts for 15% of the total cell RNA. The functional form of tRNA has a cloverleaf conformation and includes double-stranded regions. The activity of the bacillar RNases in relation to tRNA is higher than dsRNA, probably due to their ability to hydrolyze loop regions more efficiently than the stem structures.

### 2.3. Comparison of Interaction of RNases with Inhibitor Protein

**Figure 5:**
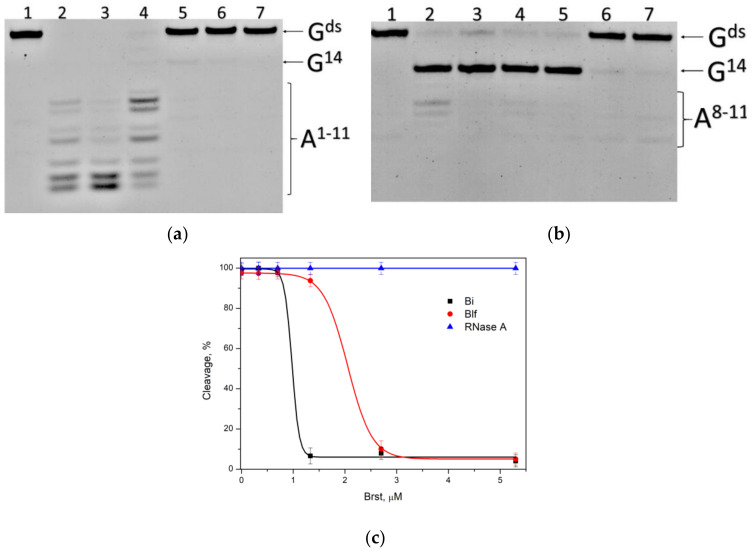
Electrophoretic separation of reaction products upon cleavage of the G^ds^ substrate by homologous RNases in the presence of barstar. (**a**) Reaction performed by binase, (**b**) Reaction performed by balifase. Lanes: (1) G^ds^ + 5.3 мкM barstar; (2) G^ds^ + Enzyme; (3) G^ds^ + Enzyme + 0.3 мкM barstar; (4) G^ds^ + Enzyme + 0.7 мкM barstar; (5) G^ds^ + Enzyme + 1.3 мкM barstar; (6) G^ds^ + Enzyme + 2.7 мкM barstar; (7) G^ds^ + Enzyme + 5.3 мкM barstar. Substrate concentration was 1 µM, enzyme concentration was 0.7 µM. (**c**) Dependence of the degree of the G^ds^ degradation by binase (Bi), balifase (Blf) and RNase A on the barstar concentration.

While inside the cell, the secreted RNases must be inhibited to avoid their toxic action. *B. amyloliquefaciens* produces a specific inhibitor of its RNase barnase termed barstar. Barstar is a small protein (10 kDa) which tightly binds to barnase at a 1:1 ratio [48]. It also inhibits binase activity in vitro, but with much lower affinity [26]. The own natural inhibitor of binase has not yet been found. We have compared the effect of barstar on the catalytic activity of binase and balifase using the model G^ds^-substrate (Figure 5). When added to the reaction mixture, barstar inhibited binase and balifase and did not affect the activity of pancreatic RNase A. The enzyme to barstar ratio, at which the complete inhibition of the ribonuclease was achieved, was 1:2 for binase and 1:4 for balifase (Figure 5). The concentration of the half-maximal inhibition IC_50_ was 1 μM for binase and 2.1 μM for balifase. We assumed that such differences relate to the quaternary structure of homologous RNases rather than their tertiary structures, since topology of their active centers is highly similar.

**Figure 6:**
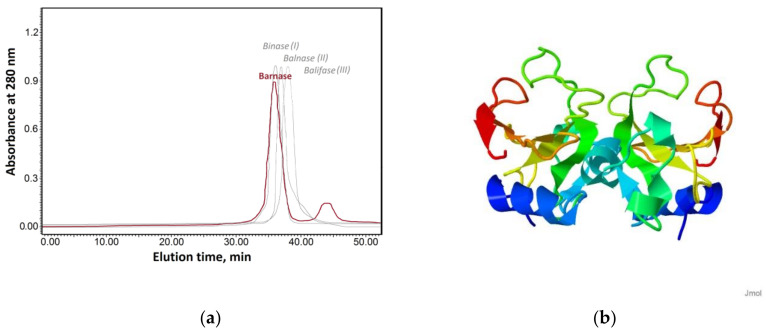
The structural organization of barnase. (**a**) Size exclusion chromatography of barnase in comparison to binase (I), balnase (II) and balifase (III). Major peaks correspond to dimer form of RNases, minor peak is represented by barnase monomer. Molecular weights of barnase peaks were calculated using equation obtained from elution profile of marker proteins. (**b**) Putative model of barnase dimer.

Binase and balifase are known to form dimers [29,30]. They differ in the mode of dimerization and stability [11,21]. The dimers of barnase were reported earlier only as a minor peak (3–6%) during its gel filtration analysis [41]. To check this fact, we have analyzed it using size-exclusion chromatography (Figure 6a). The elution profile of barnase consisted of two peaks. The first fraction was collected between 35 and 40 min which corresponds to the 25 kDa protein, and the second small peak was eluted between 40 and 45 min corresponding to the 14 kDa protein. The both peaks possessed RNase activity. No activity was detected in other fractions, confirming that barnase exists in solution in two forms, dimeric and monomeric, with the dimeric form being prevalent (Figure 6a). The modeling of the barnase dimeric structure suggested that it is likely to be formed by the interaction of N-terminal parts of its monomers (Figure 6b).

**Figure 7:**
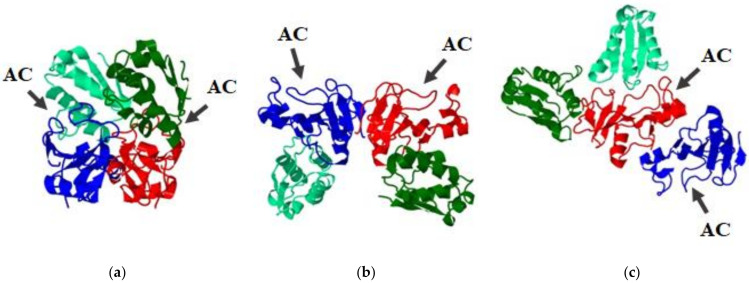
Models of barstar interaction with RNase dimers. (**a**) Barnase dimer; (**b**) balifase dimer; (**c**) binase dimer. Monomers of RNase dimers are colored in blue and red. Their active sites are faced upwards (except for the monomer of binase colored in blue). Two molecules of barstar are depicted in light and dark green. Active centers (AC) of RNases are marked by arrows.

Since RNases are dimers and it is known that the activity of barnase is blocked by barstar at a 1 to 1 ratio, for docking we used dimeric structures of RNases as receptors and two barstar molecules as ligands. We have found that in the barnase dimer, both active centers are blocked by barstar, in the binase dimer, only one of two active centers is bound by the barstar, and in the balifase dimer, both active centers are free of an inhibitor (Figure 7). As it was shown by the crystallographic studies, the barstar inhibits barnase by sterically blocking its active site with an a-helix and adjacent loop [49]. The interaction is stabilized by the electrostatic forces between the positively charged amino acid residues Lys27, Arg59, Arg83 and Arg87 of barnase, and the negatively charged Asp35, Asp39 and Glu76 of barstar [49]. Since these residues are conservative among guanyl-preferring RNases of *Bacillus*, one can assume that the same surfaces will be involved in the interaction between homologous RNases and barstar. Indeed, this is true for monomeric forms of RNases. However, dimers of RNases differ in their conformation that possibly creates novel binding interfaces. The modeling data support and help to explain the results obtained by the FRET assay (Figure 5). The predicted model of the binase–barstar interaction demonstrates that barstar cannot effectively bind both active centers of the binase dimer at a 1 to 1 ratio as in the case of barnase (Figure 5a,c); therefore, for complete binase inhibition, more molecules of barstar are required (1:2 ratio in FRET assay). The quaternary structure of balifase interfere with the binding of barstar to the active center of the enzyme (Figure 5b); therefore, the inhibition of balifase activity is possible only at a high balifase-barstar ratio (1:4 ratio in FRET assay).

## 3. Materials and Methods

### 3.1. Enzymes

Homogeneous protein preparations of RNases from *B. pumilus* (binase), *B. altitudinis* (balnase), *B. licheniformis* (balifase) and *B. amyloliquefaciens* (barnase) were obtained using previously described three-step procedure [19,30].

### 3.2. Barstar Cloning and Purification

Genomic DNA of *B. amyloliquefaciens* was extracted using Genomic DNA Purification Kit (Thermo Scientific, Waltham, MA, USA) according to the manufacturer’s protocol. Barstar gene was amplified from genomic DNA by polymerase chain reaction using Pfu DNA polymerase (Thermo Scientific, Waltham, MA, USA), forward (5′-TAGTCGGCTCAGCGTTTCCATATTGTTCATCTCC-3′) and reverse (5′-TACTCGCTCGAGATGAAAAAAGCAGTCATTAAC-3′) oligonucleotides, which included BlpI and XhoI restriction sites (underlined), correspondingly, for the subsequent cloning of barstar gene into the pET15b expression vector (Novagen, Madison, WI, USA). The purified PCR product as well as pET15b vector were digested with BlpI and XhoI restriction endonucleases and ligated. Chemically competent cells of *Escherichia coli* JM109 were transformed with the resultant plasmid pET15b-brst. Recombinant clones were selected on Luria-Bertani agar plates containing 100 μg/mL ampicillin. The integrity and correctness of the insert on pET15b-brst plasmid were checked by PCR, restriction analysis and Sanger sequencing.

For protein purification, the expression strain of *E coli* BL21 (λDE3) was transformed with pET15b-brst plasmid. Cells were grown with shaking on Luria-Bertani media containing 100 μg/mL ampicillin at 37 °C until the OD_590_ reached 0.4–0.8 units. Then 0.5 mM IPTG was added and cells were further grown for 3 h. Cells were harvested by centrifugation at 5000× *g* for 10 min. The cell pellet was resuspended in the binding buffer (1 M NaCl, 0.1% Triton X-100, 0.02 M imidazole, 15% glycerol) and sonicated 10 times for 20 sec on ice bath. Lysed cells were centrifuged at 12,000× *g* at 4 °C for 30 min. Purification of barstar from the cell-free supernatant was done using NGC Chromatography System (Bio-Rad, Hercules, CA, USA) using Ni-NTA Profinity IMAC resin (Bio-Rad, Hercules, CA, USA) equilibrated with the binding buffer. The bound enzyme was eluted with the elution buffer (0.02 mM sodium phosphate, 0.5 M NaCl and 100 mM imidazole). The collected fractions were desalted on Bio-Gel P-6 column (Bio-Rad, Hercules, CA, USA) and analyzed by SDS-PAGE in 16% polyacrylamide gel with 0.1% SDS.

### 3.3. Oligoribonucleotides

Oligoribonucleotides were obtained via the solid-phase phosphoramidite methods on the ASM-800 synthesizer (Biosset, Novosibirsk, Russia) using the corresponding phosphoramidites of N-protected 2′-O-tert-butyldimethylsilyl (2′-O-TBDMS) ribonucleotides (Chemgenes, Wilmington, MA, USA). The 5′-end of oligoribonucleotide was tagged by fluorescein residue using fluorescein phosphoramidite (Glen Research, Sterling, VA, USA). Oligonucleotides bearing a fluorescence quencher BHQ1 at the 3′-end were obtained using a modified 3′-BHQ-1 CPG (Black Hole Quencher) support (Glen Research, Sterling, VA, USA). Oligoribonucleotides were deblocked under standard mild conditions using 30% NH_4_OH in water at ambient temperature for 16 h. The 2′-O-TBDMSi protecting groups were removed by a freshly prepared NMP-Et_3_N-Et_3_N · 3HF mixture (1.5:0.75:1, *v/v/v*) at 65 °C with stirring for 1.5 h, followed by ethoxytrimethylsilane treatment and precipitation of oligoribonucleotides with diethyl ether. Deblocked oligoribonucleotides were isolated by denaturing 15% PAGE. The optical density of oligonucleotide solutions was measured on a NanoDrop 1000 spectrophotometer (Thermo Scientific, Waltham, MA, USA) relative to deionized water. The molar extinction coefficient of oligoribonucleotides or their conjugates at 260 nm was used to calculate the oligonucleotide concentrations in the initial solution. The model of oligoribonucleotides’ sequences are shown in Table 2.

### 3.4. Stopped Flow Kinetic Studies

The kinetics of the enzymatic process was registered through the change in a fluorescence intensity of the fluorescein residue, which is a part of the model substrates shown in Table 2. The fluorescence kinetic curves were recorded on a stopped-flow spectrometer SX.18MV (Applied Photophysics, Leatherhead, UK). The efficiency of the fluorescence resonance energy transfer (FRET) between the FAM/BHQ1 pair was measured by exciting the fluorescence of the FAM dye at 494 nm. The FRET signal was recorded at wavelengths above 530 nm using an OG 515 optical filter (Schott, Mainz, Germany). The instrument dead time was 1.38 ms. Each kinetic curve was averaged over at least four experimental curves. All the experiments were carried out in a buffer solution containing 50 mM Tris-HCl (pH 8.5), 50 mM KCl, 1 mM EDTA, 1 mM DTT and 9% glycerol at 25 °C. The enzyme and G^loop^ substrate were used in concentration of 0.5 μM.

For calculation of the enzymatic reaction rate constants, each kinetic curve was approximated by the sum of exponents according to the Equation (1) using the OriginPro 8.0 software package (OriginLab Corp., Northampton, MA, USA):

(1) $$F=\sum A\times e^{-k_{obs}t}+C$$

where *F* is the fluorescence intensity of FAM at time *t*; *A* is the coefficient; *k_obs_* is the determined rate constant; *C* is the fluorescence intensity at *t* → ∞.

### 3.5. Kinetic Analysis of RNA Substrates Hydrolysis by RNases in the Presence of Barstar

A kinetic analysis of the cleavage of G^ds^ substrate by binase, balifase or RNase A at the presence of barstar was carried out using the following procedure. Ten microliters of the enzyme (0.7 μM) and barstar (0.33–5.3 μM) mixture in a buffer solution were added to 10 μL of buffer solution containing the substrate (1.0 μM) and Brst (0.33–5.3 μM). After the reaction mixture had been rapidly stirred for a set period of time (5 min for Bi, 30 min for Blf and RNase A), the reaction was stopped by adding 20 μL of the solution containing 9 M urea and 25 mM EDTA, and incubated for 2 min at 96 °C. SDS-PAGE was performed in a Protean II xi vertical thermostated chamber (Bio-Rad Laboratories, Inc., Hercules, CA, USA) at 50 °C and 200–300 V. The gel was visualized using an E-Box CX.5 TS gel documentation system (Vilber Lourman, Collégien, France). The substrate cleavage efficiency was determined using Gel-Pro Analyzer 4.0 (Media Cybernetics, Rockville, MD, USA). The cleavage efficiency was calculated as the ratio of the peak area of the cleavage product to the sum of peak area of the product and the initial oligoribonucleotide. The error in determining the modification extent was usually ≤20%.

### 3.6. CD-Spectrometry

CD spectra of RNases were measured in the 190–260 nm wavelength range on the Jasco J-1500 spectrometer (Jasco, Tokyo, Japan) as described in [47]. The secondary structures of the enzymes were determined at DichroWeb server using CDSSTR algorithm and reference set of protein spectra SP175 [50].

### 3.7. Determination of RNase Activity by Acid-Soluble Products of RNA Hydrolysis

The catalytic activity of RNases was measured against yeast high-polymeric and transfer RNA, and double-stranded RNA extracted from *E. coli* (Vector-Best, Novosibirsk, Russia) using a SmartSpec Plus Spectrophotometer (Bio-Rad, Hercules, CA, USA), as described earlier [7]. One unit was defined as the amount of enzyme that increases the extinction of acid-soluble products of RNA hydrolysis at 260 nm per min at 37 °C. The specific activity was calculated as the enzymatic activity per 1 mg of protein.

### 3.8. Dynamic Light Scattering Particle Size and Zeta Potential Analysis

The average particle size and size distribution of RNases were determined by dynamic laser scattering (DLS) technology using Zetasizer Nano ZS (Malvern Instruments Ltd., Malvern, UK). Particle size was presented by volume distribution, and size distribution was evaluated by polydispersity index (PDI). The solutions were suitably diluted with ultrapure water, filtered through 0.22 nm filter and sonicated for 2 min to form a uniform dispersion before placing the sample into a quartz cuvette. Measurements were performed by ten consecutive runs on individual sample, 30 s each. The zeta potential of RNases was determined by electrophoretic light scattering technique. A diluted sample of RNase in water was allowed to stabilize at 25 °C and was placed in clear disposable zeta cells. The electrophoretic mobility between the electrodes was converted to zeta potential based on Smoluchowski equation.

### 3.9. Bioinformatic Analyses

#### 3.9.1. Prediction of Protein Secondary Structures

The DeepCNF (Deep Convolutional Neural Fields) algorithm at Raptor X server was used to predict secondary structure, solvent accessibility, and disorder regions in RNase proteins (http://raptorx.uchicago.edu/StructurePropertyPred/predict/ (accessed on 16 September 2021)). Visualization of secondary structure predictions was performed with the help of 2dSS webserver (http://genome.lcqb.upmc.fr/2dss/index.html (accessed on 20 September 2021)) and Inkscape software version 1.0.1 (https://inkscape.org (accessed on 20 September 2021)).

#### 3.9.2. Modeling of RNase Dimeric Structures and Their Interaction with Barstar

The modeling of protein–protein interaction was performed by the direct method through a search for structures with minimum Gibbs free energy using the ClusPro server [51]. The algorithm classifies predicted models into groups based on the forces involved in the protein complex formation (electrostatic, van der Waals and electrostatic, hydrophobic, or their balance). The dimer of barnase was modeled from the PDB structure 1a2pa with the help of Multimer Docking option. The structure with the lowest free energy from the balanced cluster was chosen. For binase and balifase, previously modeled structures were used [29,30]. The docking of the two barstar molecules (PDB 3da7d) into RNase dimers was performed on the ClusPro server under Receptor–Ligand mode using dimer as a receptor and barstar as a ligand. The top-ranked structures from the Electrostatic cluster were chosen. Jmol: an open-source Java viewer for chemical structures in 3D (http://www.jmol.org/ (accessed on 6 October 2021)) was used to visualize 3D structures.

### 3.10. Statistical Calculations

Each experiment was performed in triplicates. All data are presented as the mean ± standard deviation of the mean (SD). Statistical tests were performed using GraphPad Prism 8 software (GraphPad Software, San Diego, CA, USA).

## 4. Conclusions

RNases are considered as perspective therapeutics due to their biological effects. In this study, we have compared structural and functional features of homologous guanyl-preferring RNases from Bacillus, in order to address the point of whether distinctions in non-core amino acid residues can account for the dissimilarities in the structural organization of these proteins and their interaction with different molecules. We have identified that the activity levels of homologous RNases towards natural RNA substrates are similar and decrease in the row high-polymeric RNA >>> transport RNA > double-stranded RNA. However, kinetic studies on model RNA substrates have demonstrated that the cleavage rates of these enzymes are different and they are inhibited by barstar with diverse efficiency. Thus, despite the fact that RNases display the conservation of a number of active-site residues involved in substrate and inhibitor interactions, their binding rate constants differ significantly. Circular dichroism analysis has revealed significant distinctions in secondary structures of homologous RNases that can affect protein folding and oligomerization, as proved by the dynamic light scattering assay. Therefore, minor changes in structure elements of homologous proteins have a potential to significantly affect molecule structural organization, and stability and functional activities, such as catalysis or ligand binding. It should be taken into account for the developing of novel biotherapeutics based on homologous RNases since their biological effects, efficiency and safety can be changed.

## Figures and Tables

**Table 1:** Catalytic activity of RNases towards high-polymeric RNA (hpRNA), transport RNA (tRNA) and double-stranded RNA (dsRNA). Activity of each RNase towards hpRNA was taken as 100%.

	Specific RNase Activity (opt.units/mg of Protein),%		
	hpRNA	tRNA	dsRNA
Binase	100	36.3 ± 5.0	23.1 ± 1.8
Balnase	100	36.5 ± 6.5	23.7 ± 0.7
Balifase	100	31.2 ± 6.5	26.6 ± 1.7

**Table 2:** Model RNA substrates used in the work.

G^loop^	FAM-r(AUAUAAGAUCAUUAUAU)-BHQ1	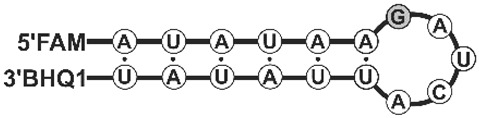
G^ds^	FAM-r(AUACAACAUAAUU**G**UAU)-BHQ1	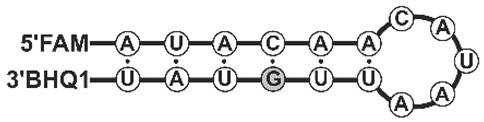

**Table 3:** Rate constant values for the interaction of binase and balifase with G^loop^ and G^ds^ substrates.

k_obs_, с^−1^	G^loop^	G^ds^
Binase	2.4 ± 0.1	0.73 ± 0.05
Balifase	0.28 ± 0.04	0.025 ± 0.002

## Data Availability

The data presented in this study are available on request from the corresponding author.
